# Supplementary material for: Backup transcription factor binding sites protect human genes from mutations in the promoter
Source: PLoS One. 2023 Aug 31;18(8):e0281569. doi: 10.1371/journal.pone.0281569 (PMC10470901; doi:10.1371/journal.pone.0281569)
Supplement: S1 Table — a Most abundant transcription factor binding site (i.e., rank 1 transcription factor binding site). b ChIP-seq signal for rank 1 transcription factor binding site. c Gene expression in TPM from GTEx. (DOCX) [file pone.0281569.s001.docx]

Supplementary Table 1: All human brain-specific genes used in this study (42 genes)

| gene | tfbs1^a^ | ChIP1^b^ | tfbs2 | ChIP2 | tfbs3 | ChIP3 | tfbs4 | ChIP4 | tfbs5 | ChIP5 | exp1^c^ |
| --- | --- | --- | --- | --- | --- | --- | --- | --- | --- | --- | --- |
| ANKRD34C | REST | 2231 | CTCF | 1526 | EHMT2 | 1158 | EZH2 | 1000 | HDAC2 | 1000 | 6.3 |
| ANKRD63 | HNRNPL | 1691 | HDAC2 | 1577 | CTBP1 | 1505 | KDM4A | 1478 | L3MBTL2 | 1327 | 4.4 |
| BARHL1 | RNF2 | 1743 | SUZ12 | 1670 | REST | 1479 | EZH2 | 1000 | HDAC1 | 1000 | 49.1 |
| BHLHA9 | EZH2 | 735 | HDAC6 | 604 | KDM4A | 460 | GABPA | 460 | YY1 | 310 | 1.3 |
| BTBD17 | ZEB1 | 1499 | ZNF143 | 1393 | CTCF | 1199 | GABPA | 1000 | NR2C1 | 1000 | 6.3 |
| C4orf50 | CTCF | 16230 | RAD21 | 11686 | KDM1A | 7288 | ZNF143 | 6682 | MXI1 | 6400 | 9.7 |
| CACNG8 | SIN3A | 3143 | SP1 | 3019 | JUND | 2848 | POLR2A | 2755 | KDM4A | 2372 | 39.4 |
| CALHM1 | USF | 900 | GATAD2B | 539 | ZNF263 | 399 | MAX | 376 | ZBTB33 | 340 | 3.1 |
| CREG2 | FOS | 3219 | CTCF | 2482 | MAFK | 2071 | MAFF | 1734 | RAD21 | 1509 | 51.9 |
| EN2 | RNF2 | 2924 | EZH2 | 1000 | SUZ12 | 1000 | ZNF263 | 1000 | L3MBTL2 | 614 | 68 |
| FGF3 | EZH2 | 1410 | SUZ12 | 1079 | KDM4A | 998 | GATA2 | 795 | RXRA | 775 | 25.1 |
| FREM3 | EP300 | 3009 | CTCF | 2017 | RUNX3 | 2000 | FOS | 1688 | NR2F2 | 1586 | 1.5 |
| GBX2 | RNF2 | 2248 | POLR2A | 1580 | E2F6 | 1387 | EGR1 | 1330 | USF1 | 1000 | 2.4 |
| GPR101 | KDM4A | 1319 | EZH2 | 1244 | NRF1 | 1000 | SUZ12 | 865 | L3MBTL2 | 647 | 4.1 |
| GRIN2B | CTCF | 11057 | SP1 | 11020 | EP300 | 10847 | JUND | 9741 | RAD21 | 8550 | 6.9 |
| GRM4 | CTCF | 12716 | RBM25 | 6865 | KDM1A | 6510 | ZMYM3 | 6404 | RAD21 | 6323 | 286.8 |
| GSX1 | REST | 1185 | EZH2 | 1000 | RNF2 | 1000 | SIN3A | 1000 | SUZ12 | 1000 | 1.2 |
| HCRT | IKZF1 | 1236 | L3MBTL2 | 1000 | MAX | 1000 | MGA | 1000 | MYC | 1000 | 38.4 |
| HTR5A | REST | 1394 | MXI1 | 1101 | TRIM28 | 1000 | XRCC5 | 1000 | ZBTB33 | 1000 | 35.5 |
| HTR6 | KDM1A | 2254 | REST | 2126 | SIN3A | 1579 | EMHT2 | 1177 | NRF1 | 1051 | 4.4 |
| KCNK4 | EZH2 | 3748 | SUZ12 | 2806 | RNF2 | 1691 | KDM4A | 1167 | ZKSCAN1 | 1000 | 14.1 |
| KCNV1 | KDM4A | 1604 | EZH2 | 1473 | HDAC2 | 1190 | CTCF | 1147 | SUZ12 | 944 | 16 |
| LCN8 | ZMTM3 | 1347 | CTCF | 1094 | BCOR | 1000 | KDM1A | 1000 | SETDB1 | 1000 | 35.6 |
| MEPE | FOS | 3594 | CTCF | 2188 | STAT3 | 2079 | RAD21 | 1870 | SMC3 | 1210 | 4.9 |
| MINDY4B | EP300 | 1791 | FOS | 1681 | CEBPB | 1493 | GATA3 | 1468 | CBX8 | 1436 | 2.1 |
| NKX6-3 | KDM4A | 1000 | RBBP5 | 1000 | ASH2L | 933 | EZH2 | 856 | HDAC6 | 721 | 12.8 |
| OTP | RNF2 | 4000 | PAX5 | 2071 | SUZ12 | 2000 | RBBP5 | 1894 | RUNX3 | 1823 | 9.4 |
| PABPN1L | AGO2 | 1668 | PCBP1 | 766 | FIPL1 | 762 | RBFOX2 | 657 | POLR2G | 541 | 3.8 |
| PNMA6F | KDM4A | 670 | ZEB2 | 284 | RFX1 | 91 | PKNOX1 | 63 | CBFA2T3 | 28 | 26.1 |
| POU6F2 | SMARCA4 | 16814 | EP300 | 11208 | CTCF | 10901 | MAFK | 7561 | EZH2 | 6618 | 1.9 |
| PTH2 | ZBTB33 | 1791 | KDM1A | 1241 | CTBP1 | 1000 | CTCF | 1000 | EZH2 | 1000 | 2.2 |
| RHO | SP1 | 1060 | CEBPB | 1000 | ASH2L | 722 | RBBP5 | 710 | CTBP1 | 653 | 0.5 |
| RTP1 | NRF1 | 448 | EP300 | 293 | ZNF263 | 202 | ZNF639 | 172 | RFX1 | 169 | 3.6 |
| SLC35D3 | EZH2 | 939 | HDAC6 | 905 | KDM4A | 801 | SUZ12 | 627 | E2F6 | 492 | 5.8 |
| SLC6A3 | EZH2 | 7848 | MNT | 2712 | CTCF | 1593 | HDAC2 | 1573 | ATF2 | 1472 | 34.7 |
| SSTR4 | KDM4A | 987 | EZH2 | 625 | ZNF263 | 537 | SUZ12 | 457 | GABPA | 317 | 1.3 |
| STH | ATF2 | 1000 | ATF7 | 1000 | JUND | 671 | CREB1 | 633 | BCOR | 495 | 2 |
| TBR1 | EZH2 | 4000 | RNF2 | 3000 | SUZ12 | 3000 | CBX2 | 2542 | CHD1 | 2105 | 42 |
| TLX3 | RNF2 | 1816 | RBBP5 | 1071 | TBP | 1068 | EZH2 | 1000 | ZNF263 | 1000 | 48.3 |
| TMEM132D | FOS | 14312 | EP300 | 12091 | CTCF | 11202 | ATF2 | 8464 | KDM1A | 8448 | 9 |
| VAX1 | RNF2 | 3000 | CHD1 | 1191 | KDM4A | 1188 | EZH2 | 1000 | SUZ12 | 1000 | 1.6 |
| ZP2 | CEBPB | 1974 | RNF2 | 1000 | HNF4A | 907 | GATA2 | 748 | FOXA2 | 669 | 66.4 |

^a^ Most abundant transcription factor binding site (i.e., rank 1 transcription factor binding site).

^b^ ChIP-seq signal for rank 1 transcription factor binding site.

^c^ Gene expression in TPM from GTEx.
