# Supplementary material for: Backup transcription factor binding sites protect human genes from mutations in the promoter
Source: PLoS One. 2023 Aug 31;18(8):e0281569. doi: 10.1371/journal.pone.0281569 (PMC10470901; doi:10.1371/journal.pone.0281569)
Supplement: S2 Table — a Most abundant transcription factor binding site (i.e., rank 1 transcription factor binding site). b ChIP-seq signal for rank 1 transcription factor binding site. c Gene expression in TPM from GTEx. (DOCX) [file pone.0281569.s002.docx]

Supplementary Table 2: All human liver-specific genes used in this study (31 genes)

| gene | tfbs1^a^ | ChIP1^b^ | tfbs2 | ChIP2 | tfbs3 | ChIP3 | tfbs4 | ChIP4 | tfbs5 | ChIP5 | exp^c^ |
| --- | --- | --- | --- | --- | --- | --- | --- | --- | --- | --- | --- |
| AFP | POLR2A | 9187 | RBM22 | 8670 | RBFOX2 | 2310 | FOXA2 | 1858 | FOXA1 | 1819 | 1.3 |
| AGXT | HNRNPL | 4336 | RXRA | 4203 | HNF4A | 4200 | MAX | 3624 | SP1 | 3575 | 1731.5 |
| AHSG | RBM22 | 5881 | POLR2A | 5374 | AGO2 | 4172 | RBM39 | 3775 | SRSF4 | 3394 | 1346.5 |
| C8A | HNF4A | 8689 | EP300 | 7435 | JUND | 5913 | MAX | 5901 | FOXA1 | 5489 | 274 |
| C8B | HNF4A | 5517 | SP1 | 4501 | FOXA2 | 2983 | RXRA | 2968 | FOXA1 | 2579 | 295.2 |
| C9 | HNF4A | 5403 | RXRA | 4667 | SP1 | 4659 | TAF1 | 4198 | YY1 | 3673 | 404.9 |
| CA5A | AGO2 | 8657 | RBM22 | 6267 | CTCF | 5422 | RAD21 | 3852 | POLR2A | 3659 | 6.4 |
| CFHR2 | CBX5 | 597 | RELB | 472 | RUNX3 | 451 | BATF | 444 | NFIC | 368 | 298.7 |
| CFHR3 | TAF1 | 1104 | RXRA | 641 | HNF4A | 612 | YY1 | 335 | POLR2A | 321 | 109.6 |
| CFHR4 | CTCF | 1623 | RAD21 | 1583 | YY1 | 1221 | HNF4A | 1199 | FOXA1 | 1140 | 24.6 |
| CFHR5 | YY1 | 1719 | FOXA2 | 1534 | RXRA | 1509 | HNF4A | 1318 | JUND | 1162 | 39.2 |
| CYP26A1 | HDAC2 | 1790 | ZBTB33 | 1535 | RXRA | 1117 | NCOR1 | 1000 | TAL1 | 1000 | 4.5 |
| CYP7A1 | JUND | 1240 | FOXA2 | 1089 | ARID3A | 1000 | FOXA1 | 1000 | HNF4A | 1000 | 2.6 |
| F13B | RUNX | 1080 | FOXA1 | 1044 | RAD21 | 995 | RAD21 | 893 | TAF1 | 789 | 29.8 |
| F2 | RBM22 | 7303 | AGO2 | 6184 | RBFOX2 | 5977 | POLR2A | 5356 | POLR2G | 4776 | 599.7 |
| F9 | HNF4A | 2685 | SP1 | 2607 | RXRA | 2378 | FOXA2 | 2294 | FOXA1 | 1759 | 235.2 |
| FGF21 | RXRA | 2491 | SP1 | 1790 | HNF4A | 1449 | POLR2A | 1413 | EP300 | 1177 | 18.7 |
| GBP7 | YY1 | 2699 | HNF4A | 2609 | SP1 | 1646 | MAFK | 1355 | FOXA1 | 1285 | 26.7 |
| GDF2 | RNF2 | 1946 | UBTF | 1785 | MNT | 1681 | MAX | 1449 | L3MBTL2 | 1431 | 8.3 |
| INHBC | HNF4A | 3056 | MAX | 2507 | EGR1 | 2311 | RXRA | 2128 | NR2F2 | 2111 | 37.7 |
| INS-IGF2 | CTCF | 3290 | RAD21 | 3109 | SMC3 | 2863 | MAX | 1653 | RXRA | 1377 | 7.4 |
| LPA | SMARCA | 2280 | CTCF | 2226 | ATF2 | 2052 | YY1 | 1687 | ATF7 | 1635 | 15 |
| MBL2 | RXRA | 3565 | FOXA1 | 2313 | TAF1 | 2282 | FOXA2 | 2247 | EP300 | 2000 | 61 |
| RTP3 | RXRA | 2048 | CEBPB | 1523 | HNF4G | 1191 | HNF4A | 1187 | SP1 | 968 | 24.2 |
| SERPINA7 | RBFOX2 | 1458 | RAD21 | 1136 | CTCF | 1000 | HNF4A | 1000 | MAFF | 1000 | 51.3 |
| SLC17A2 | HNF4A | 4963 | FOXA1 | 3322 | EP300 | 2969 | FOXA2 | 2960 | SP1 | 2607 | 34.5 |
| SLC22A10 | FOXA1 | 1231 | HNF4A | 1000 | FOXA2 | 914 | FOSL2 | 868 | ARID3A | 846 | 9.4 |
| SLC22A25 | FOXA2 | 3197 | SP1 | 3121 | HNF4A | 2491 | EP300 | 2221 | FOS | 2172 | 17 |
| SPP2 | HNF4A | 1984 | RXRA | 1386 | JUND | 1374 | HNRNPK | 1259 | SP1 | 1196 | 65.9 |
| UGT2B10 | FOXA2 | 2469 | RP300 | 2036 | FOXA1 | 1690 | CREM | 1383 | RBFOX2 | 1263 | 110.9 |
| UROC1 | SP1 | 4222 | CTCF | 3664 | RAD21 | 2591 | HNF4A | 2229 | HNRNPL | 2065 | 75.6 |

^a^ Most abundant transcription factor binding site (i.e., rank 1 transcription factor binding site).

^b^ ChIP-seq signal for rank 1 transcription factor binding site.

^c^ Gene expression in TPM from GTEx.
